# Including climate change in community-based obesity prevention interventions: a qualitative exploration of the perspectives of Australian funders

**DOI:** 10.1186/s12889-025-22599-2

**Published:** 2025-04-24

**Authors:** Nicole Ward, Kim Robinson, Jane Jacobs, Melanie Nichols, Marj Moodie, Vicki Brown

**Affiliations:** 1 Deakin Health Economics, Institute for Health Transformation, Deakin University, Geelong, Australia; 2 Global Centre for Preventive Health and Nutrition, Institute for Health Transformation, Deakin University, Geelong, Australia; 3 School of Health and Social Development, Deakin University, Geelong, Australia

**Keywords:** Obesity, Climate change, Funding, Community-based obesity prevention interventions, Double-duty actions

## Abstract

**Background:**

Community-based obesity prevention interventions (CBOPIs) demonstrate promise as effective, cost-effective approaches to prevent obesity. Whilst CBOPI actions often focus on obesity-related outcomes, they may also have positive impacts on climate change. Actions that simultaneously address obesity and climate change are known as double-duty actions. For example, switching to active modes of transport benefits individual health, while also reducing emissions from vehicle use. Support from CBOPI funding decision-makers is crucial for intervention success; the factors influencing funding decisions are currently not well understood. This study aimed to identify factors that influence funding decisions within organisations, to determine whether funders recognise double-duty actions in CBOPIs, and which double-duty actions are preferred.

**Methods:**

Potential participants with CBOPI funding decision-making roles were purposively sampled and invited to participate. Potential interview participants from government and non-government organisations were identified by search engine (Google) and invited via email to partake in an interview. Sixty-five invites were emailed and seven interviews with eight participants were conducted between April–May 2023. The participating stakeholders all had health roles; four State-wide and four local government. Semi-structured interviews with eight participants were conducted over Zoom between February-May 2023. Interviews were transcribed using Zoom Transcription and analysed with the assistance of NVivo. Reflexive Thematic Analysis underpinned the data analysis and the Social Ecological Model was used to further develop the theory.

**Results:**

Results suggested that participants recognised double-duty actions and believed inclusion of climate change action in CBOPIs would improve both intervention outcomes and participant acceptability. However, participants believed that stringent funding models limit flexibility to include climate change action. This could be mitigated by incorporating climate change into strategic health plans. Community partnerships may also be an effective tool to enhance double-duty actions in CBOPIs, as they allow participants to tailor interventions to community concerns including climate change.

**Conclusion:**

CBOPIs that use double-duty actions to intentionally target obesity prevention and climate change action may play an important role in addressing two critical public health issues at the community level. Whilst CBOPI funders are supportive of double-duty actions, modifications from strategy and partnerships may be required to realise the successful implementation.

**Supplementary Information:**

The online version contains supplementary material available at 10.1186/s12889-025-22599-2.

## Background

Obesity carries a significant disease burden due to its high prevalence and contribution to causing, or worsening, many disease states [1]. Obesity was once viewed as solely the responsibility of the individual and a product of individual behavioural choices. Now obesity is understood to be a modifiable consequence of modern society and systems [2]. Community-based obesity prevention interventions (CBOPIs) use a socio-ecological lens to address modifiable risk factors through a variety of actions across multiple settings [3, 4]. A socio-ecological lens considers how factors beyond the individual influence health and behaviours and articulates how policies, systems and community resources can influence and affect health [4, 5].

### Community-based obesity prevention interventions

CBOPIs are defined as multi-component and multi-setting interventions, designed to actively target and engage a minimum of two different community sectors (e.g. family/ household, media, community/ recreation centres, schools, after school activities, local business, local government) [6]. Key features of successful CBOPIs are the incorporation of community capacity building and the engagement of community stakeholders [7, 8]. CBOPIs have shown moderate success in reducing the prevalence of obesity, particularly childhood obesity, however the sustainability of CBOPIs and their long-term impact on obesity prevalence are challenging to measure [3, 9–11].

The primary and secondary outcomes of CBOPIs are usually measured by anthropometry (e.g. body mass index), diet (e.g. serves of fruit per day) and/ or physical activity (e.g. minutes spent being active/ week). Additional broader benefits that are not an intended outcome are considered co-benefits. Co-benefits may arise from the intervention’s implementation (e.g. community capacity building), or can be benefits that are additional to the primary obesity prevention objective of the intervention (e.g. a CBOPI targeting children may also improve the health of adults in the same residence) [12]. Measuring co-benefits may generate a more accurate and comprehensive picture of the impact of CBOPIs across communities. For instance, the Gardeneers was a CBOPI that aimed to increase fruit and vegetable consumption [13]. This study also collected data on the social and emotional co-benefits of the intervention, such as self-management and social skills [13]. Co-benefit outcomes are less likely to be studied (using either qualitative or quantitative methods), may only be anecdotally recognised or may not be recognised explicitly at all [14]. This may be due to a range of factors, including researchers’ perspectives of which co-benefits are most important, finite resources with which to conduct evaluations and consideration of time and cognitive burden required to complete data collection [15–17]. Climate change, like obesity, is a serious public health issue and together they form a global syndemic [2]. Syndemics are formed by concurrent epidemics that share drivers and related outputs [2]. The important links between obesity and climate change have only been recognised relatively recently and currently there is limited evidence on how they interact [2, 18]. Actions that simultaneously impact obesity prevention and climate change are known as double-duty actions [2]. For example*,* energy-dense and nutrient-poor sugar sweetened beverages (SSBs) are commercially produced and distributed using large amounts of energy, and excessive packaging. Reducing SSB intake may reduce obesity prevalence through a reduction in kilocalories consumed, whilst reducing the green-house gas emissions associated with their production, distribution and disposal of packaging. Studies focused on double-duty actions are a rapidly emerging field, as exemplified by The Lancet’s Countdown on Health and Climate Change series and other key recent publications on the global syndemic [2, 18–20].

### Double-duty actions

Double-duty actions in CBOPIs are implemented with the primary intention to reduce the prevalence of obesity, though some may also offer the potential broader benefit of improving climate change [14, 15]. Double-duty actions have been identified as occurring in CBOPIs. It is plausible that if an intervention is effective and the effect is sustained over time CBOPIs may result in climate change benefits. Currently, this is hypothetical as limited evidence exists on how interventions targeting obesity, like CBOPIs, might implicitly or explicitly impact on climate change and other environmental adversities [2, 18]. To assist different stakeholders to recognise and explicitly incorporate double-duty actions in CBOPIs, the DoublE-duty actions in CommunIty-baSed obesity InterVEntions (DECISIVE) framework was developed [21]. A systematic approach was taken to identify and classify double-duty actions to build the framework. The framework details nine double duty action areas, plus a final *Other* category to capture additional actions in this emerging area [21]. For each action area, the framework provides descriptions on the potential impact on both obesity and climate and example community-based implementation strategies for each double-duty action. DECISIVE may be applied to CBOPIs by stakeholders, including funders, implementers and policy makers, to recognise if interventions have elements of climate change action, and, if these elements can be promoted so CBOPIs can simultaneously address both important issues to improve public health [2].

### Community-based obesity prevention funding

Funding decision-makers may have a role in deciding which CBOPI interventions are funded for implementation in the community. Funding community-based interventions is complex due to competition for resource allocation, limited setting-specific evidence for the effectiveness of CBOPIs, varying community needs and evolving government priorities [8, 22–24]. Funders, who are usually from government or non-government organisations must consider community needs, policies, existing and required resources, intervention effectiveness and feasibility of implementation in the community, as well as the political and social landscape [23, 25].

In Australia, community-based health interventions are usually resourced through state funding models [26]. Ananthapavan et al. [11] and Lui [27] have both studied the application of economic evaluation by prevention decision-makers, however to date there is limited research on what motivates and influences funding decisions specifically in relation to CBOPIs. Given the multiple considerations and limited resources for allocation, decision-makers with funding influence have a challenging yet pivotal role in the implementation and continuity of CBOPIs [25]. Whilst the literature indicates that stakeholders see the benefits of incorporating climate change action in CBOPI [28], it is unclear if funders consider climate change in their decision-making process and how this influences other factors they must consider when allocating resources.

Currently, to our knowledge, there is no literature on funder preferences for CBOPIs so it is unknown whether stakeholders with funding responsibilities recognise the potential impact that CBOPIs may have on climate change and if so, how they view the addition of climate change benefits. This study therefore seeks to determine if stakeholders with funding capacity in CBOPIs, recognise the potential for including double-duty actions. Additionally, it also seeks to understand whether the inclusion of double-duty actions changes how funders perceive the benefits of CBOPIs, and to ascertain which double-duty actions are preferred. The findings will contribute to a better understanding of what funders perceive as beneficial to CBOPIs, both in the context of climate change action and more broadly.

## Methods

The COnsolidated criteria for REporting Qualitative research (COREQ) checklist for reporting qualitative research was used to ensure standardised qualitative reporting [29]. Semi-structured interviews were conducted with funders or potential funders of CBOPIs. This study did not include research funding decision-makers. Individual interviews were used instead of focus groups to provide an opportunity for greater understanding of the influences on stakeholders’ decision-making and their perceptions [29]. Participants were recruited via purposive sampling based on their own or their organisation’s role in funding CBOPIs. Organisations that fund CBOPIs were identified via an internet search (Google) and researchers’ knowledge. State and local governments and agencies were approached as they most commonly fund CBOPIs in Australia so were identified as the most suitable participants. An interview invitation was emailed to the identified organisations’ general administration addresses, unless an individual’s email address could be publicly sourced. The invitation detailed the interview topics and indicated that the interview would be held over Zoom for no longer than 60 min. Complete knowledge of funding allocation processes and financial jurisdiction was not a requirement to participate. Interviewees were not screened for their level of funding knowledge and any participant agreeing to be interviewed was included in the study. Participants from government and non-government organisations at state and local levels, from the eight Australian states and territories were invited to participate, with the aim of providing a broad range of views. Snowball sampling was also used, with participants asked to suggest additional participants both via return email and during the interview.

Interview invitations were emailed in February to April 2023. If no response was received, a single follow-up email invitation was sent one week after the original invitation. For stakeholders who agreed to participate, interviews were scheduled at a mutually agreeable time with an interviewer experienced with CBOPI design and delivery (NW). Prior to interview each participant provided their consent to participate in the study via returned signed consent form. Interviews were conducted online using Zoom software (San Jose, CA, Zoom Video Communications Inc).

Interview questions and techniques were developed to ensure consistency was maintained across the interviewees. An interview schedule (see Additional file 1, Appendix 1) was developed by the lead author (NW) and refined with the research team. The schedule was based on current literature in the field, including the work developing the DECISIVE framework. The interview schedule aimed to understand if funders and potential funders recognise interventions as containing double-duty actions and how funders perceive double-duty actions as being viewed by different stakeholders or community groups (e.g. parents or older persons). Participants were asked questions about their experience with CBOPIs; factors considered in funding decisions; prior consideration of additional co-benefits of CBOPIs; recognition of double-duty actions; perceived effectiveness of double-duty actions; perceived community engagement and acceptability of double-duty actions; and if the inclusion of double-duty actions would affect funding decisions. The nine double-duty actions as identified by the DECISIVE framework, and example strategies for each double-duty action, were emailed to participants prior to the interview for their reference (Table 1).

**Table 1:** Participant interview handout of DECISIVE double-duty actions and example strategies

**Double-duty actions**	**Example strategies**
Eat locally	• Consume fresh foods • Purchase seasonal fruit and vegetables that have less food miles • Promote local food markets and ensure local markets are accessible to all community members
Eat more fruit and vegetables	• Price promotion for fruits and vegetables • Local government funding for school fruit and vegetable gardens • Nude food days at schools and workplaces
Eat less ultra-processed food (UPF)	• Restrict access to fast food • Healthy food swaps • (swap energy dense packaged foods for fresh alternatives) • Healthy catering; avoid reliance on UPFs for workplace events
Eat less meat and more plant-based protein	• Cooking classes, recipes and demonstrations for meat alternatives • ‘Meat-free’ days • Plant-based protein choices on menus
Reduce consumption of sugar sweetened beverages	• Price, promotion and placement of water to increase intake • Encourage reusable drink bottles • Ensure free drinking water is widely available at clubs
Improve education opportunities	• Facilitate education on traditional Indigenous diets • Add health and climate change into curriculum including renewable energy
Reduce screen time	• Promote active play • Encourage incidental physical activity
Modal shift from motorised to active transport	• Local council to install and maintain footpaths in high pedestrian traffic areas • Promote and encourage cycling through safe cycling infrastructure and campaigns • Encourage public transport use with regular services and competitive pricing
Improve green space	• Local government community planning to incorporate green space in urban planning • Local funding for community gardens
Other	

Nude foods days: Events to encourage and support the consumption of fresh and unpackaged foods e.g. fruit. Other double-duty action: This category was intentionally added to accommodate additional double-duty actions that may be identified through the testing and development of DECISIVE in this study and beyond, as the health and climate change field continues to evolve

All interviews were transcribed using the Zoom automated transcription function and checked for accuracy and consistency by the lead researcher (NW). Each participant was provided with the interview transcript to check and given the opportunity to redact any information from the interview. A dual-staged coding system using the six stages of Reflexive Thematic Analysis [30] was used by two researchers (NW and JJ) to analyse the data, using Nvivo software (Lumivero, Denver, USA). In the initial stage, semantic codes were developed by NW using the research questions. A second stage of coding was then undertaken using Reflexive Thematic Analysis to deduce semantic and then latent codes [30] by NW and JJ. Further code development was refined through discussions with the research team, where discrepancies were resolved.

Finally, the Social Ecological Model was applied to review and interpret the data. A social ecological approach considers how social environments influence individuals’ choices and behaviour [5, 31] making it appropriate for the CBOPI community setting. The model was used as it identifies the constructs within the community, such as policies, infrastructure and inter-organisational relationships, that influence an individual’s health. The Social Ecological Model encapsulates these broad areas that may influence health outcomes whilst maintaining agency to the individual [5, 31]. This mirrors the approach CBOPIs use as they implement supportive, environmental modifications that influence the individual. This inductive and deductive approach to data analysis enabled the participants’ views on double-duty actions and on how they potentially influence funding decisions to be understood. The process then allowed for broader themes that were important to participants to be identified. Quotes from interview participants were included to accurately illustrate their viewpoints [29].

## Results

Sixty-five potential participants were invited via email, with eight agreeing to participate. No data was collected about those who declined to participate or did not reply. All of the interviews, conducted between February and May 2023, were with single participants, except for one interview where two participant colleagues were interviewed together. Six participants were recruited from direct email invitations and two via purposive sampling. Stakeholders were from state-wide (*n* = 5), or local health roles (*n* = 3). There were two interviewees each from the states of Victoria, Queensland and South Australia, and one each from New South Wales and Tasmania. All interviewees held government funded health positions and none were responsible for direct CBOPI delivery. Interview durations ranged from 28 to 38 min. When participants were provided with the interview transcripts, four provided minor amendments.

The overall finding of the study was that participants believed double-duty actions could be explicitly incorporated into CBOPIs and that this would enhance the benefits of the intervention, however the inclusion of double-duty actions may not influence funding decisions. The analysis of the interviews identified five themes as outlined below.

### Theme one: Climate change as part of CBOPI

All participants were able to recognise double-duty actions from the provided list and most had considered the ability of CBOPIs to influence climate change. Participants felt promoting the co-benefits of climate change action in CBOPIs would be beneficial and that the intervention communities would be interested and receptive to actions that benefit climate change. Some actions, such as modal shift from motorised to active transport, were more readily recognised as double-duty actions than others, such as improving green space.

Participants were motivated and engaged with explicitly promoting double-duty actions in CBOPIs as they believed community members were also connected with these messages.

*“Climate is going to be so closely connected with nutrition and obesity prevention, and it’s going to become a bigger and bigger focus. It's also what we heard during our [community] consultation” Participant 6.*

*“Eat locally kind of action is a strong one. Especially with people who are passionate about the environment. And passionate about their state” Participant 8.*

Among the identified double-duty actions, participants felt that most would be reasonably acceptable to communities, but there were notable variations in the expected acceptability across categories. Participants believed that community members would value the *‘Eat locally’* double-duty action the most. They also felt *‘Increase fruit and vegetables’* and *‘Improve education opportunities’* would be most accepted by the community, particularly in schools and by parents. However, participants felt the action of *‘Reducing ultra-processed foods (UPFs)’* would not be as readily accepted by all members of the community, as messaging around restricting foods is not well received compared to messaging around encouraging foods and activities.

*“It's politically palatable to promote fruit, vegetables and water but it's less easy to get action on restricting access to these things [UPFs]. So we get less traction on that kind of activity” Participant 4.*

*‘Promoting active transport’* was deemed another acceptable action; however, some participants raised that infrastructure constraints (e.g., availability of regular, inexpensive public transport), environmental factors (weather and adequate shade) and safety concerns could limit actionability. ‘*Eat less meat and more plant-based protein’* was the action thought by participants least acceptable to community members as they believed most Australians enjoyed a meat-containing diet. Whilst participants appeared confident to suggest which double-duty actions would be accepted by communities in general, participants found it challenging to speculate which double-duty actions would be more acceptable to different population sub-sets, for example school children or retirees.

Overall, participants believed that most members of the community were motivated to some degree by climate change action and felt the incorporation of health and climate change was well regarded by both health professionals delivering CBOPIs and the community. Participants believed the incorporation of double-duty actions in CBOPIs would enhance the benefits of an intervention.

### Theme two: Strategy as a priority

The importance of aligning a CBOPI to their organisation’s strategies was a crucial consideration for participants. Participants explained that funding is usually provided to fulfil a set of objectives that have been set by policy-makers. CBOPI funding is generally derived from obesity prevention strategies. Funders must ensure the intervention and the evaluation of the intervention meet the original obesity-focused objectives set by policy and the organisation’s strategic plan.

*“We prioritise, based on our strategic plan and what our strategic goals are” Participant 7.*

Objectives and benefits that were perceived to be outside of the strategy, like climate change, were unable to be considered by funders even if they felt there was value in the intervention.

*“Funding has to come through a certain source, and that is often tied to a certain priority ……if all the participants are …enjoying just being outdoors and meeting and making friends, and that's all really valuable stuff, but it doesn't necessarily help you…” Participant 2.*

*“How it is valued [would change] if those benefits aligned to the strategic priorities… we often find [if it is] broader than just obesity that potential providers will come to us with a program but because they are not clear on our priorities we find it challenging to fit the program within the funding buckets” Participant 1.*

Participants believed it would be beneficial to include climate change in CBOPIs. However only funders whose work was guided by an overarching strategy that included climate change (such as the Victorian Public Health and Wellbeing Plan 2019–2023 [24]) felt that they were able to consider explicitly incorporating, implementing and promoting climate change actions into their CBOPIs.

*“It really depends on the objective of the organisation. So if our organisation had signed up to the sustainable development goals it would make an intervention more attractive [to fund]” Participant 1.*

Where climate change objectives were not included in their strategy or policies, participants expressed they were not able to prioritise interventions based on potential climate change impacts even if they believed community participants would engage more with climate change objectives. Participants saw opportunity in presenting the double-duty actions of an intervention as this would allow climate change actions to be promoted without deviating from the original obesity prevention messaging. Generally, participants did not believe they had the flexibility to modify programs in a manner that best met the immediate needs of their communities if this was not aligned to their organisation’s strategy or policy. Participants whose organisations had a strategic plan that included climate change were more likely to consider including climate action in CBOPIs.

*“Commissioning has to be done within quite tight restrictions that the Commonwealth set out….it can mean that we have less flexibility” Participant 1.*

### Theme three: Importance of effectiveness

Participants noted it was essential that they could be confident an intervention would be effective in their community setting before supporting its funding. As the primary objective of CBOPI is obesity prevention, participants were interested in obesity-related effectiveness for their communities. Participants believed limited resourcing meant funders were unlikely to fund interventions where there was little or no demonstrated evidence of success. There were mixed views from participants as to the level of evidence required for decision-making. Some participants would consider anecdotal evidence whereas others required objective evaluations based on results from the intervention community or a community with a similar demographic.

*“Evidence, or…promising even anecdotal evidence of what might be effective for a particular place-based approach to a particular community” Participant 2.*

*“There needs to be some evidence of effectiveness” Participant 6.*

*“Everything we do is evaluated…it really is important… everything we drive is based on evidence” Participant 8.*

It was, however, also acknowledged by some participants that an intervention must be appropriate for their specific community setting and positive evaluation from another setting may not necessarily mean an intervention was appropriate for their communities; due to differences in community infrastructure (for example, resourcing), demographics or population needs.

If a newer intervention had not had the opportunity to demonstrate effectiveness, some participants may still consider supporting it. However, it would not be prioritised over existing programs and would be more likely to be considered if it aimed to meet a need in the community that was not currently being met by other established programs.

*“Some evidence, or promising even anecdotal evidence of what might be effective for a particular place-based approach to a particular community…It’s always difficult getting exactly the evidence you need in prevention” Participant 2.*

Interventions that promoted double-duty actions could be considered but they would be assessed on the effectiveness of the obesity component. Climate change co-benefits were viewed as a valuable addition but were seen as a “bonus” benefit. As double-duty actions were not included in strategic priorities, they were not subjected to the same critical evaluation that the obesity component of the CBOPI would be.

### Theme four: Forming community partnerships

The formation of partnerships was sought and highly valued by all participants. Participants reported that the rigidity of funding models where funding was linked to organisational strategies (as described above), and the disconnect between strategies and perceived imminent community needs, were catalysts for the formation of community alliances and partnerships. Partnerships were viewed as a vehicle to promote objectives such as climate change that were perceived as valuable yet sat outside of an organisation’s own strategy. Participants observed that the intent behind the intervention could be different for different organisations. For example, in a community garden initiative some organisations valued climate change action, some obesity prevention, and some mental health, yet all worked together by pooling resources and promoting the intervention to successfully establish a community garden.

All participants reported benefits from forming community alliances and partnerships to enhance community interventions. Partnerships were formed after previous successful collaboration on different projects or if the organisational objectives aligned and the intervention was unable to be funded by one organisation.

*“Bringing the local service providers together so that they're supporting each other and working together. We're actually seeing quite an increase in the number of events and activities being offered” Participant 6.*

*“We just started thinking how we could work together* [with council] *and we just shared…and now we have a* [partnership] *website and extra volunteers” Participant 3.*

Partnerships allowed resources, including funding, to be shared amongst several organisations so additional objectives could be met while maintaining the integrity of the original funding. This is particularly important for CBOPIs as it allows co-benefits such as climate change to be promoted. The inclusion of local governments in partnerships was seen as instrumental for success of community interventions, as local governments could drive local infrastructure changes and were actively engaged with different stakeholder groups. One participant noted how their local government was the main driver for a “greening” initiative.

*“So we've been working with the Council, and with a whole lot of other stakeholders, and we've been successful in getting a grant ...” Participant 5.*

The "greening” idea was initiated by a researcher who was working in partnership with the community and presented the health benefits of climate change to the local group. Through the formation of partnerships with researchers, obesity-focused groups, climate change focused groups, local health organisations, local council, government departments and other local groups, they are currently investigating how they can implement “greening” to simultaneously benefit obesity and climate change.

Partnerships were seen as a solution to overcoming funding silos and single objectives such as obesity prevention, whilst optimising resource allocation to implement CBOPIs that best meet the needs of the community. This was achieved by enabling funding, existing resources and community connections to be pooled together. Another benefit of partnerships and alliances was that double-duty actions and additional health messaging were shared to wider audiences. Therefore, forming partnerships and alliances were seen as an effective tool to overcome funding restraints and strategy rigidity whilst strengthening community messaging through cross-promotion of interventions.

*“We need all the communities to come together to change the environment that we work in” Participant 5.*

*“It can get people more engaged in their community, in a social sense or even just to incorporate some walking or team sports or gym or something um that activity gets you more connected to the community as well” Participant 1.*

### Theme five: Negative connotations of obesity

Participants emphasised that they avoid using the term ‘obesity,’ given it is negatively viewed by community members and some health professionals. Interventions labelled as obesity prevention were seen as punitive, and participants believed community members would be discouraged from participating in interventions which included the term ‘obesity’.

*“People don't necessarily want to come along to something where you're talking about obesity” Participant 2.*

*“We can get push back from community…. about using the word obesity” Participant 3.*

To navigate this challenge of community members being *“nervous or suspicious”* (Participant 1) of obesity interventions, obesity interventions were commonly framed to focus on the positive benefits of the intervention for general wellbeing. Interventions with an explicit focus on increasing physical activity, improving nutrition and better sleep hygiene (as opposed to an explicit focus on addressing obesity) were considered a solution to reduce the negative connotations associated with the term ‘obesity’.

*‘We might use that language [obesity] internally ... But…community framing is generally about well being’ Participant 4.*

Explicitly promoting the climate change benefits of double-duty actions presents another opportunity to increase the positive intervention benefits without using obesity terminology given the focus on climate change. Furthermore, given the community interest in climate change the promotion of double-duty actions may enhance the CBOPI’s community engagement.

Notably, some participants commented on the positive language selected for use in the DECISIVE framework. The language of encouragement and promotion of positive behaviour change used in DECISIVE, instead of providing prescriptive instructions, was viewed as being achievable and therefore consistent with messaging that would be better received by the community.

## Discussion

This study sought to determine if stakeholders with CBOPI funding decision-making capacity recognised double-duty actions in CBOPIs for their dual obesity prevention and climate change action roles, and if so, how the inclusion of double-duty actions alters their perceptions of CBOPIs. The study also investigated community preferences for double-duty actions from the interview participants’ viewpoints.

Participants were able to identify actions commonly undertaken as part of CBOPIs as double-duty and were motivated to incorporate double-duty actions in CBOPIs. The benefits of promoting double-duty actions were clear to participants and they were aware of the benefits for both obesity prevention and climate change action. Participants were aware of the needs of their intervention communities and were committed to ensuring interventions met these needs. The participants understood that climate change action was an important issue for many in their communities. However, despite this awareness of community concerns, participants found it more challenging to identify which DECISIVE double-duty actions would be most accepted by particular groups within their communities.

### Influences on funding decisions

This study found the main influences on funding decisions were organisational strategy and policy, and evidence of effectiveness of interventions. This aligns to research that indicates that intervention effectiveness, political priorities, community values as well as decision-makers beliefs influence funding decisions [32, 33]. Funding was channelled to align with strategy and measures of intervention effectiveness. The primary objective of the strategies was obesity prevention, therefore funding and intervention effectiveness were focused on obesity related outcomes. To simultaneously incorporate obesity prevention and climate change action benefits in CBOPIs, organisational strategies need to enable both of these significant issues to be explicitly funded and targeted with CBOPIs. To date, there is little evidence of this occurring [34]. Notably some strategies, such as the Victorian Health and Wellbeing Plan, do incorporate climate change objectives [35] so there is potential for more organisations to add climate change objectives to health strategies. An overarching health strategy that incorporates climate change would allow stakeholders to include climate change objectives in CBOPIs. Double-duty actions were seen as an advantageous method to include climate change action in obesity prevention interventions to increase the benefits from an intervention. Funding for CBOPIs with double-duty actions could be secured through obesity objectives if participants could be confident on intervention effectiveness related to obesity prevention.

### Views on double-duty actions

The presentation of the double-duty actions was raised as an important consideration by participants. Participants suggested that actions that proposed restricting foods or activities were likely to be met with resistance from community members. Actions aimed at restricting unhealthy behaviours were reported to be viewed by community members as removing their personal choice or as constituting a criticism of their health status*.* This is reflected in the literature where it is reported that health promotion programs can be perceived by the community as dictating behaviour [15]. The inclusion and promotion of the benefits of climate change in CBOPIs may showcase the benefits for others and therefore enhance community members’ willingness to modify behaviour and implement the ‘restrictive type’ actions. Further research is required about whether community members are more motivated to change behaviour in CBOPIs if they see the primary purpose of this behaviour change as benefiting climate change. However, due to strategy and funding constraints participants viewed climate change actions as extra benefits to CBOPI as opposed to a critical part of the CBOPI.

### Tension from policy for community

Participants’ reliance on being answerable to organisational strategies to set priorities and subsequently fund interventions may create tension in their decision-making as participants may want to prioritise other interventions that they believe will better meet the individual needs of their communities. Whilst strategies were broad and rarely specific to individual communities, participants sought to ensure interventions were compatible with the individual needs of their communities. This generates an increasingly challenging space to navigate for funders as communities become more diverse and guidance is provided by multiple policies [24]. This may mean funded CBOPIs need to be modified in certain communities to meet their cultural or linguistic needs. Alternatively, recruitment strategies may need to be altered to better engage different communities. Furthermore, the needs that a community identifies as important may be incongruent to the priorities identified by health strategies. Participants felt that climate change action was valued by communities, however they felt that the tensions from competing factors in strategic plans impacts on how funders can prioritise double-duty actions.

### Partnerships

A solution to this tension was found in partnerships. Partnerships enable organisations to share resources and coordinate interventions, which optimise resource allocation and generates more support allowing organisations to “do more” [15, 36, 37]. All participants noted the value they have observed and/or experienced from the formation of partnerships. The strongest example of this was a multi-agency double-duty intervention of greening where different community groups with different agendas and resources came together to achieve multiple outcomes. For organisations whose strategy did not articulate certain priorities including climate change, forming partnerships allowed these priorities to be addressed. This provided the flexibility to meet community needs without compromising on strategy intent which was an important protector in order to secure future funding. This is supported by the literature which suggests that considering the individuality of local communities is crucial for the success of CBOPIs and that positive collaboration is key to driving change [4, 15, 36, 38]. Furthermore, partnerships create greater intervention visibility in the community which is vital for intervention success [4]. For double-duty actions this means the intervention is reaching both obesity prevention and climate change focused groups. Whilst participants spoke of the gains they experienced from partnerships, the formation of partnerships were decribed as serendipitous as opposed to intentional. Partnerships with formalised agreements have strong outcomes including effectiveness and community transformation [37]. The OPAL CBOPI in South Australia attributed gains to building successful partnerships and subsequently a recommendation from the evaluation was skills training to foster community partnerships [15]. This creates an opportunity for potential partnerships to be formally identified in communities and actively built.

### Discussion summary

The needs of the community were prioritised by participants when making funding decisions. The assessment of evidence as relevant to their communities, negative connotations of obesity, and climate change in CBOPI were all themes that were derived from perceived individual community needs or community. Whilst strategies provided direction, it is important that interventions can be molded to fit to the needs of communities [24].

Participants worked to ensure resources were well used, and valued interventions that they believed to be effective in their communities. This is consistent with the literature which reiterates the importance of intervention evidence and evaluation for funding and policy decision-making [4]. Where the intervention evidence was not from a similar community to the participant’s, participants sought flexibility in program implementation to be able to meet the needs of their community. Flexibility to make funding decisions that benefit the local community, like explicitly incorporating double-duty actions is essential as stronger outcomes are achieved where communities are empowered to adapt interventions to their own needs [4, 8]. Interestingly, participants were supportive of climate change objectives in CBOPIs although the evidence for double-duty actions is still emerging [2]. This may be because participants felt the climate change actions were a “bonus” to the health outcomes of CBOPIs, and that climate change actions matter to their communities.

Participants noted that the negative connotations of the term ‘obesity’ influenced how CBOPIs are delivered. Much of the work in CBOPIs is focused on a more general concept of wellness through improved nutrition, physical activity and sleep as opposed to a direct reduction in obesity or other chronic disease risk factors. This may be related to the stigma associated with obesity, where obesity is viewed as a personal failing [2] and the participants’ awareness that further stigmatisation could have adverse impacts on communities [39]. Presently much of the work around obesity prevention is not combined with other non-health objectives such as climate change [2]. Introducing double-duty actions as an explicit focus in CBOPI may present a way to promote behaviour changes that may have double-duty impacts, without focusing on obesity. This concept of motivating obesity-related behaviour change through the societal benefits of climate change has been documented by Thomas Robinson as “stealth interventions”. The proposal is that interventions that focus on the benefits of intervention participation or outcomes unrelated to obesity may be more engaging for communities than interventions focused on obesity-related outcomes [40]. Whether this results in greater engagement from the community warrants further investigation. The participants saw benefits in including and promoting the co-benefits of CBOPIs including climate change and believed climate change action was valued by many members of their local communities. This concept is supported by Patrick and Kingsley [28] who found community health practitioners are motivated to incorporate climate change actions into the health agenda.

### Strengths and limitations

A key strength of the study was the applicability of the research outcomes to policy-makers, partnerships, funders and CBOPI implementers and participants. The research identified key opportunities that each group could implement to enhance the climate change actions in CBOPI. These recommendations are presented in Table 2.

**Table 2:** Practical recommendations for stakeholders

Stakeholder group	Recommendations for each stakeholder group
Policy-makers and funders	• Include climate change objectives in obesity prevention strategies • Encourage and support formation of partnerships • Allow flexibility in CBOPI implementation • Advocate for climate change action in obesity prevention strategies • Consider strategy and community needs • Encourage and support partnerships • Identify needs of community
Community organisations and partnerships	• Create and maintain partnerships to foster climate change action in CBOPIs that meet community needs • Consider both strategy objectives and community needs when forming partnerships
CBOPI Implementers	• Advocate for climate change action in obesity prevention strategies • Seek and support opportunities for partnerships • Listen to needs of the community

Another strength was the focus on funders. Funders provide a unique lens adjunct to policy-makers and implementers. Funders were, however, a challenging group to target and some participants expressed concern about disclosing funding agreements. Others who declined to participate stated they did not feel that they had the jurisdiction to comment.

The limitations of the study were the small sample size of eight participants in health roles. As the interviews required an opt-in approach, it is possible that funders with a professional or personal interest in climate change were more likely to participate in the study. Participants were speaking about their own experiences in their professional context and were not directly asked about funding sources. However, the themes presented in the interview data were consistent across participants who worked in different settings and geographical locations, suggesting the themes are relevant to many CBOPI funders. Participants could nominate the DECISIVE actions that they felt were more acceptable to communities though they felt it harder to determine if certain population groups would be more engaged with specific double-duty actions. This may have been because participants were not implementing CBOPIs and therefore did not receive this insight from community members. Exploring the views of CBOPI participants and implementers is an area for future work. The motivation of community members to make behavior changes based on health versus climate change benefits was not clear to the decision-makers and also warrants further investigation. Finally, double-duty actions are an emerging area and there is limited evidence, particularly in the community sector. There are currently no CBOPIs that have measured the intervention’s impact on both obesity and climate change, and this is an area for significant further work.

This study has contributed important policy and practice opportunities. Double-duty actions are present in many CBOPIs and it may be practical and beneficial to simultaneously promote the benefits of obesity and climate change in CBOPI. Understanding how organisational strategy is used in funding decision-making may encourage the designers of interventions to include climate change objectives in obesity strategies. It could also act as a catalyst for those working at the community level to advocate for the inclusion of community priorities in overarching strategies. For community organisations that are positioned to form partnerships, this study has articulated the benefits of partnerships and how they can be used to achieve multiple objectives, such as aligning obesity and climate change.

## Conclusion

Funders recognised the potential climate change benefits of CBOPIs and believed climate change action to be a potential positive benefit of a CBOPI. However, the identification and promotion of double-duty actions of obesity prevention and climate change actions in a CBOPI would only influence funding decisions if it was directed by organisational strategy. Funders align CBOPIs with organisational strategies, but experience shows this may be too inflexible to meet the diverse needs of individual communities. Forming partnerships is a viable solution to overcoming this as it enables resources to be pooled and therefore can generate outcomes that meet both the funding organisation’s requirements and community needs. Finally, in order to ensure CBOPIs have the flexibility to simultaneously support obesity prevention and climate change action, climate change needs to be included as an objective in health strategies linked to obesity prevention.

## Supplementary Information

Additional file 1: Appendix 1, Interview schedule.

## Data Availability

The data used in this study is interview transcripts. These interview transcripts datasets are securely stored on a Deakin University password protected drive. To protect interview participants raw data sets are not available however the de-identified transcripts may be made available on request.
