# Supplementary material for: Including climate change in community-based obesity prevention interventions: a qualitative exploration of the perspectives of Australian funders
Source: BMC Public Health. 2025 Apr 24;25:1526. doi: 10.1186/s12889-025-22599-2 (PMC12020184; doi:10.1186/s12889-025-22599-2)
Supplement: Supplementary file 1 — Additional file 1: Appendix 1, Interview schedule. [file 12889_2025_22599_MOESM1_ESM.docx]

Additional file

Interview schedule

**ETHICS APPROVED**

**Semi-structured interview guide: PRECIS: PRecision Evidence for Childhood obesity prevention Interventions – Economic stream**

General/ Overview questions

1. Would you please tell me about your experience with childhood community-based obesity prevention interventions?

2. What factors are considered when deciding to fund CBIs? Which of these factors are the most important to consider?

1. Let’s talk about the key health benefits that you see are a result of community-based obesity prevention interventions first? From your experience, what would you say are the health benefits of CBIs, if any?
2. Now I would like you to think more broadly, about whether you think there are any other benefits from community-based obesity prevention interventions? So, benefits that aren’t necessarily to do with health.

Can you tell me what some of those benefits might be, if any?

1. Usually when a community-based obesity prevention interventions is proposed or introduced to a community, the identified and promoted benefits relate to only to the primary objective, ie, obesity prevention or physical health. Do you think identifying and promoting co-benefits (that is the broader benefits of a CBI), alongside the health benefits may change the engagement or how the CBI is valued. Please consider this from the perspective of:
    Individual children
    Households and families
    Communities as a whole

Double-duty actions

Climate change is a co-benefit of some CBIs actions. Actions that potentially influence both obesity and climate change are called double-duty actions.

6. Please have a look at the list of actions provided [provide list]. Can you identify the double-duty actions from this list?

7. The list you have been provided are all double-duty actions. Which double-duty actions do you think are most accepted by different stakeholder groups to include in community-based obesity interventions? [To provide list of double-duty actions]

Funding

8. If obesity prevention and climate change action were included together as a specific objective of a CBI, would this affect funding decisions?

9. Prior to this study have you previously considered the additional co-benefits from CBIs?

Thank you for your time today. Those are all the questions I have. Would you like to add anything else?
